# Feature weight estimation for gene selection: a local hyperlinear learning approach

**DOI:** 10.1186/1471-2105-15-70

**Published:** 2014-03-14

**Authors:** Hongmin Cai, Peiying Ruan, Michael Ng, Tatsuya Akutsu

**Affiliations:** 1 School of Computer Science and Engineering, South China University of Technology, Guangdong, China; 2 Institute for Chemical Research, Kyoto University, Kyoto, Japan; 3 Department of Mathematics, Hong Kong Baptist University, Hong Kong, China

**Keywords:** Feature weighting, Local hyperplane, Classification, RELIEF, KNN

## Abstract

**Background:**

Modeling high-dimensional data involving thousands of variables is particularly important for gene expression profiling experiments, nevertheless,it remains a challenging task. One of the challenges is to implement an effective method for selecting a small set of relevant genes, buried in high-dimensional irrelevant noises. RELIEF is a popular and widely used approach for feature selection owing to its low computational cost and high accuracy. However, RELIEF based methods suffer from instability, especially in the presence of noisy and/or high-dimensional outliers.

**Results:**

We propose an innovative feature weighting algorithm, called LHR, to select informative genes from highly noisy data. LHR is based on RELIEF for feature weighting using classical margin maximization. The key idea of LHR is to estimate the feature weights through local approximation rather than global measurement, which is typically used in existing methods. The weights obtained by our method are very robust in terms of degradation of noisy features, even those with vast dimensions. To demonstrate the performance of our method, extensive experiments involving classification tests have been carried out on both synthetic and real microarray benchmark datasets by combining the proposed technique with standard classifiers, including the support vector machine (SVM), *k*-nearest neighbor (KNN), hyperplane *k*-nearest neighbor (HKNN), linear discriminant analysis (LDA) and naive Bayes (NB).

**Conclusion:**

Experiments on both synthetic and real-world datasets demonstrate the superior performance of the proposed feature selection method combined with supervised learning in three aspects: 1) high classification accuracy, 2) excellent robustness to noise and 3) good stability using to various classification algorithms.

## Background

Feature weighting is an important step in the preprocessing of data, especially in gene selection for cancer classification. The growing abundance of genome-wide sequence data made possible by high-throughput technologies, has sparked widespread interest in linking sequence information to biological phenotypes. However, the expression data usually consist of vast numbers of genes (≥10,000), but with small sample size. Therefore, feature selection is a necessary for solving such problems. Reducing the dimensionality of the feature space and selecting the most informative genes for effective classification with new or existing classifiers are commonly adopted techniques in empirical studies.

In general, the feature weights are obtained by assigning a continuous relevance value to each feature via a learning algorithm by focusing on the context or domain knowledge. The feature weighting procedure is particularly useful for instances based on learning models, in which a distance metric is typically constructed using all features. Moreover, feature weighting can reduce the risk of overfitting by removing noisy features, thereby improving the predictive accuracy. Existing feature selection methods broadly fall into two categories: wrapper and filter methods. Wrapper methods use the predictive accuracy of predetermined classification algorithms (called base classifiers), such as the support vector machine (SVM), as the criterion for determining the goodness of a subset of features [1,2]. Filter methods select features according to discriminant criteria based on the characteristics of the data, independent of any classification algorithms [3-5]. Commonly used discriminant criteria include entropy measurements [6], Fisher ratio measurements [7], mutual information measurements [8-10], and RELIEF-based measurements [11,12].

As a result of emerging needs in the biomedical and bioinformatics fields, researchers are particularly interested in algorithms that can process data containing features with large (or even huge) dimensions, for example, microarray data in cancer research. Therefore, filter methods are widely used owing to their efficient computation. Of the existing filter methods for feature weighting, the RELIEF algorithm [13] is considered to be one of the most successful owing to its simplicity and effectiveness. The main idea behind RELIEF is to iteratively update feature weights iteratively using a distance margin to estimate the difference between neighboring patterns. The algorithm has been further generalized (with the new algorithm referred to as RELIEF-F) to average multiple nearest neighbors, instead of just one, when computing sample margins, whose name is RELIEF-F [13]. Sun et al. showed that RELIEF-F achieves significant improvement in performance over the original RELIEF. Sun also systematically proved that RELIEF is indeed an online algorithm for a convex optimization problem [11]. By maximizing the averaged margin of the nearest patterns in the feature scaled space, RELIEF can estimate the feature weights in a straightforward and efficient manner. Based on the theoretical framework, I-RELIEF, an outlier removal scheme, can be applied since the margin averaging is sensitive to large variations [11].

To accomplish sparse feature weighting, the author incorporated a *l*_1_ penalty into the optimization by I-RELIEF [12].

In this paper, we propose a new feature weighting scheme within the RELIEF framework. The main contribution of the proposed algorithm is that the feature weights are estimated from local patterns approximated by a locally linear hyperplane, and thus we call the proposed algorithm as LH-RELIEF or (LHR), for short. It is shown that the proposed feature weighting scheme achieves good performance when combined with standard classification models, such as the support vector machine (SVM), naive Bayes (NB) [14], *k*-nearest neighbors (KNN), linear discriminant analysis (LDA) [15] and kierarchical *k*-nearest neighbor (HKNN) [16]. The superior performance with respect to classification accuracy and excellent robustness to data heavily contaminated by noises make the proposed method promising for using in bioinformatics, where data are severely degraded by background artefacts owing to sampling bias or the high degree of redundancy, such as in the simultaneous parallel sequencing of large/huge numbers of genes.

The advantages of our method are as follows: (1) The gene selection process considers the discriminative power of multiple similar genes that are conditional on their linear combinations. This allows joint interactions between genes to be fully incorporated to reflect the importance of similar genes; (2) LHR assigns weights to genes and thus allows the selection of important genes that can accurately classify samples; (3) Using the genes selected by LHR, classic classifiers including NB, LDA, SVM, HKNN and KNN achieved comparable or even superior accuracy as reported in the literature. This confirms that incorporation of interactions among similar genes in feature weighting estimation under local linear assumptions not only conveys information of the underlying bio-molecular reaction mechanisms, but also provides high gene selection accuracy.

## Results and discussion

To evaluate the performance of the proposed LHR, we conducted extensive experiments on different datasets. First, we performed experiments on a synthetic data from the famous Fermat’s spiral problem [17]. We then tested it on nine medium to large benchmark microarray datasets, which were all used to investigate the relationship between cancers and gene expression.

### Evaluation methods

In this study, we tested the performance of the proposed LHR by combining it with standard classifiers, including NB, KNN, SVM, and HKNN [16]. We applied leave-one-out cross-validation (LOOCV) or 10-fold cross validation (CV) to evaluate classification accuracy. LOOCV provides an unbiased estimate of the generalization error for stable classifiers such as KNN. Using LOOCV, each sample in the dataset was predicted by the model built from the rest of the samples and the accuracy for each predication was included in the final measurement. Using the 10-fold CV scheme, the dataset was randomly divided into ten equal subsets. At each turn, nine subsets were used to construct the model while the remaining subset was used for prediction. The average accuracy for 10 iterations was recorded as the final measurement. For classifiers with tuning parameters (such as the SVM), the optimal parameters were first estimated with 5-fold CV using the training data and then used in the modeling. To simplify the comparison, some of the accuracy results were taken from the literature.

### Parameter settings

LHR takes two parameters: the number of nearest neighbors (*k*) and the regularized constant (*λ*). The choice of *k* depends on the sample size. For small samples, *k* should be small, such as 3 or 5, whereas for large samples, *k* should be set to a larger value, such as 10 or 20. Performance generally improves as *k* increases, however, beyond a certain threshold, larger values of *k* may not lead to any further improvement [18]. A rule of thumb is to set *k* to be the odd number 7. *λ* helps to stabilize the matrix inversion from singular and is generally a tiny constant. In our experiments, we set *λ*=10^-3^.

### Synthetic experiments on Fermat’s spiral problem

**Figure 1:**
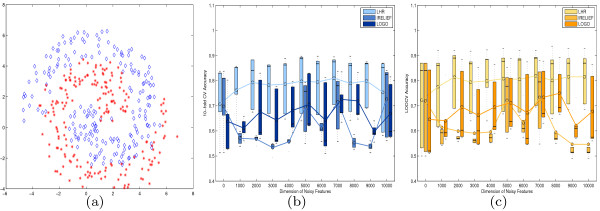
**Experiments on the Fermat’s Spiral problem.(a)** The Spiral consists of two classes, each having 200 samples labeled by different colors; Boxplot results after LHR, I-RELIEF and LOGO through five classifiers on **(a)**, degraded by noise features whose dimension extending from 0 to 10000. Two criteria of 10-fold CV **(b)** and LOOCV **(c)** are used to evaluate the performance of the feature selection methods. The result after various classifier is marked in red circle. The averaged values were connected to highlight the different performance.

In the first experiment, we tested the performance of the proposed method on the well-known Fermat’s spiral problem. The test dataset consists of two classes with 200 samples for each class. The labels of the spiral are completely determined by its first two features. The shape of the Fermat’s spiral distribution is shown in Figure 1(a). Heuristically, the label of a sample can easily be inferred from its local neighbors. Therefore, classification based on local information thus gives a more accurate result than global measurement based prediction (or classification) since the latter is sensitive to noise degradation. To test the stability and robustness of LHR, irrelevant features following the standard normal distribution were added to the spiral for classification testing. The dimensions of the irrelevant features were set to {0,1000,2000,3000,4000,5000,6000,7000,8000,9000,10000}. To compare the ability to recover informative features, both the I-RELIEF and LOGO algorithms were also used because of its intrinsic closeness to LHR. The three feature weighting schemes were first applied to rank the importance of the features. Only the top five ranked features were retained to test the robustness of feature selection schemes under noisy contamination. Performance comparisons were conducted on the truncated dataset using five classic classifiers: SVM, LDA, NB, KNN, and HKNN. For each experiment, both 10-fold CV and LOOCV were used to evaluate the classification accuracy. To eliminate statistical variations, we repeated the experiments ten times on each dataset and recorded the average classification errors. The detailed numerical results are given in Tables 1 and 2 for 10-fold CV and LOOCV, respectively. To visualize the results, we created a box plot of the distributions thereof for the experimental results after 10-fold CV and LOOCV in Figure 1(b) and (c), respectively. Each plot represents the classification accuracy for a single dataset. Figure 1(b) shows the 10-fold CV accuracy for each of the five classifiers against the dimensions of the noisy features. Figure 1(c) shows the LOOCV accuracy values against the dimensions of the noisy features. We use dark colors to denote the accuracy results achieved using I-RELIEF and LOGO, while a light color is used for those by LHR. In most cases, the performance of LHR coupled with various classifiers is superior to that of both I-RELIEF and LOGO, and thus the corresponding box plot lies above the ones for I-RELIEF and LOGO.

**Table 1:** Ten-fold CV experiments on robustness of the feature weighting on the spiral with irrelevant noisy feature

**Spiral data**	**LHR**						**I-RELIEF**						**LOGO**					
**Dimension**	**SVM**	**LDA**	**NB**	**KNN**	**HKNN**	**Aver.**	**SVM**	**LDA**	**NB**	**KNN**	**HKNN**	**Aver.**	**SVM**	**LDA**	**NB**	**KNN**	**HKNN**	**Aver.**
0	83.0	83.0	51.3	83.8	52.5	75.2	81.0	81.0	51.5	82.5	51.5	**75.4**	53.0	52.5	53.0	80.3	80.3	63.8
1000	86.0	61.2	61.8	92.0	87.0	**77.6**	59.8	53.8	55.0	57.7	59.0	57.0	56.0	59.3	60.7	63.7	65.8	61.1
2000	90.0	69.0	67.0	92.0	89.0	**81.4**	57.0	54.0	58.0	57.3	57.5	56.8	57.0	58.5	59.5	80.3	83.0	67.7
3000	87.5	67.0	64.0	91.8	86.8	**79.4**	54.5	55.0	53.3	49.5	54.5	53.3	56.3	55.0	55.8	77.8	76.8	64.3
4000	88.5	64.0	66.3	92.3	88.5	**79.9**	55.8	56.5	58.3	51.7	56.0	55.6	59.3	62.8	61.5	77.5	79.8	68.2
5000	89.0	67.8	66.8	92.8	87.8	**80.8**	79.3	59.8	55.0	75.8	78.0	69.5	62.0	62.3	62.7	83.0	82.7	70.5
6000	88.8	66.3	67.5	92.0	88.3	**80.5**	65.8	55.8	60.0	60.8	63.0	61.0	54.0	51.0	56.0	79.2	81.3	64.3
7000	89.3	69.5	70.0	92.0	89.0	**81.9**	83.8	60.8	55.8	79.7	78.0	71.6	67.3	63.2	67.3	81.5	83.5	72.5
8000	86.8	65.0	66.8	93.8	88.3	**80.1**	55.0	58.5	57.0	53.5	52.5	55.3	66.8	64.8	66.5	78.5	83.3	72.0
9000	88.8	68.5	70.8	92.8	87.0	**81.6**	56.0	51.2	53.0	54.8	54.0	53.8	64.0	59.0	61.3	55.5	58.5	59.7
10000	88.8	68.5	70.8	92.8	87.0	**81.6**	84.0	57.5	56.3	82.5	83.3	72.7	59.8	57.3	57.8	78.5	81.5	67.0

When using 10-fold CV criteria, the LHR outperforms both LOGO and I-RELIEF in terms of accuracy by classical classifier of SVM, LDA, NB, KNN and HKNN. The better averaged value after the three methods are highlighted in bold. With the increase of dimension of the irrelevant features, the performance of LHR keeps stable.

**Table 2:** LOOCV experiments on robustness of the feature weighting on the spiral with irrelevant noisy feature

**Spiral data**	**LHR**						**I-RELIEF**						**LOGO**					
**Dimension**	**SVM**	**LDA**	**NB**	**KNN**	**HKNN**	**Aver.**	**SVM**	**LDA**	**NB**	**KNN**	**HKNN**	**Aver.**	**SVM**	**LDA**	**NB**	**KNN**	**HKNN**	**Aver.**
0	84.0	53.3	50.0	87.0	87.0	**72.3**	84.0	51.7	50.0	87.0	87.0	72.0	51.0	51.5	52.0	84.3	84.3	64.6
1000	86.0	61.2	61.8	92.0	87.0	**77.6**	59.3	54.3	54.8	78.3	60.5	61.4	56.8	57.7	59.3	82.3	64.3	64.1
2000	90.0	69.0	67.0	92.0	89.0	**81.4**	56.8	57.0	55.8	72.3	57.8	59.9	57.3	57.8	60.3	88.5	83.8	69.5
3000	87.5	67.0	64.0	91.8	86.8	**79.4**	56.8	55.3	52.8	74.3	56.3	59.1	60.0	54.8	54.5	85.3	76.5	66.2
4000	88.5	64.0	66.3	92.3	88.5	**79.9**	55.5	58.8	57.3	71.3	55.3	59.6	59.0	60.5	61.8	86.5	79.5	69.5
5000	89.0	67.8	66.8	92.8	87.8	**80.8**	81.3	59.5	57.0	85.3	77.8	72.2	61.8	60.8	63.7	88.8	81.0	71.2
6000	88.8	66.3	67.5	92.0	88.3	**80.5**	64.3	57.3	59.5	74.0	61.0	63.2	54.8	54.5	57.0	87.5	82.5	67.3
7000	89.3	69.5	70.0	92.0	89.0	**81.9**	83.5	61.0	54.8	88.0	79.5	73.3	63.0	65.5	67.0	87.8	83.8	73.4
8000	86.8	65.0	66.8	93.8	88.3	**80.1**	0.0	56.5	59.3	69.8	55.0	48.1	66.5	67.5	69.3	89.3	82.5	75.0
9000	88.8	68.5	70.8	92.8	87.0	**81.6**	51.5	49.0	51.2	68.0	53.5	54.6	62.0	57.5	61.0	73.3	57.0	62.1
10000	88.8	68.5	70.8	92.8	87.0	**81.6**	51.5	49.0	51.2	68.0	53.5	54.6	57.0	57.5	56.0	86.5	81.8	67.8

When using *LOOCV* criteria, the LHR outperforms both I-RELIEF and LOGO in terms of accuracy after classical classifier of SVM, LDA, NB, KNN and HKNN. The better averaged value after the two methods are highlighted in bold. With the increase of dimension of the irrelevant features, the performance of both LOGO and I-RELIEF are degraded while LHR keeps stable.

The line graph of the average performance confirms that the proposed method is more robust to noise than I-RELIEF and LOGO. In both CV experiments, we observed that the performance of the three methods was very similar in case where the dimension of the irrelevant features was small. For example, with a zero dimension of irrelevant features, i.e, no noisy features, classification results by the five classifiers were very similar. The average accuracy is 75.2*%* for LHR and 75.4*%* for 10-fold CV, 72.3*%* for LHR and 72.0*%* for LOOCV. However, as the dimension of the irrelevant features increases, both the performance of I-RELIEF and LOGO are severely degraded by the noisy features. In comparison, the performance of LHR is very stable and superior to that of the other combinations. In both experiments, the overall accuracy by LHR is better than that of I-RELIEF and LOGO. We also observed that the accuracies after LOGO, when combining with the five classifiers, were in small variance. This nice property implies that the LOGO method could derive features that are less dependent on classification model, and thus are less redundant than LHR and I-RELIEF do.

### Empirical large/huge microarray datasets

In the second experiment, we tested the performance of the proposed algorithm on nine binary microarray datasets. The benchmark datasets, which have been widely used to test a variety of algorithms, are all related to human cancers, including the central nervous system, colorectal, diffuse large B-cell lymphoma, leukemia, lung, and prostate tumors. Characteristics of the datasets are summarized in Table 3.

**Table 3:** **Summary of the tested microarray datasets [**[19]**]**

**Dataset**	**Platform**	**Gene no.**	**Samples no.**
Colon	cDNA	2000	62
Leukemia	Affy	7129	72
CNS	Affy	7129	34
DLBCL	Affy	7129	77
Lung	Affy	12533	181
Prostate1	Affy	12600	102
Prostate2	Affy	12625	88
Prostate3	Affy	12626	33
GCM	Affy	16063	280

We note that most of the test datasets have small sample sizes (less than 100). This poses a difficulty in evaluating the performances of classifiers using the standard fold CV schemes. In this experiment, the LOOCV method was used instead to estimate the accuracy of the classifiers. Each sample in the dataset was predicted by a classifier constructed using the rest of the samples. To assess the generality of the selected informative genes, classic classifiers including LDA, KNN, NB, HKNN and SVM were tested on the selected genes. The experimental results are summarized in Table 4. Note that some of the results were taken directly from the literature.

**Table 4:** Classification accuracies (%) on 9 real data sets

**Method**	**Datasets**									**Average**
	**CNS**	**Colon**	**DLBCL**	**GCM**	**Leukemia**	**Lung**	**Prostate1**	**Prostate2**	**Prostate3**	
TSP [20]	77.90	91.10	*98.10*	75.40	93.80	98.30	95.10	67.60	97.00	88.26
k-TSP [19]	97.10	90.30	**97.40**	85.40	95.83	98.90	91.18	75.00	97.00	92.01
PAM [19]	82.35	89.52	85.45	82.32	94.03	97.90	90.89	81.25	94.24	88.66
sumdiff-PAM [21]	79.41	87.10	87.01	83.57	95.83	98.34	93.14	77.27	96.97^*γ*^	88.74
mul-PAM [21]	85.29	90.32	92.21	82.86	95.83	98.90	92.16	79.55	93.94	90.12
sign-PAM [21]	85.29	88.71	94.81	81.07	95.83	98.90	90.20	76.14	100	90.11
HBE [22]			96.10		98.61		96.08			
IVGA-SVM [23]		91.61			97.22		92.06			
BBF-SVM [24]		87.10	92.71				94.12			
SVM [25]	82.35	83.87	96.10	93.57	98.61	98.90	91.18	76.14	*100*	91.19
NB [25]	79.41	58.06	79.22	82.5	98.61	98.34	62.75	80.68	93.94	81.50
BMSF-SVM [25]	94.12	*95.16*	**97.40**	98.57	98.61	99.45	97.06	*98.86*	*100*	*97.69*
BMSF-LDA [25]	97.06	87.10	96.10	90.36	98.61	97.79	95.10	94.32	96.97^*η*^	94.82
BMSF-QDA [25]	97.06	90.32	94.81	90.36	97.22	97.23	94.12	90.91	*100*	94.67
BMSF-NB [25]	94.12	87.10	88.31	87.86	95.83	98.90	89.22	89.77	*100*	92.34
LHR-SVM^*ξ*^	*100*	87.10	94.81	*100*	98.61	*100*	96.08	**95.45**	*100*	96.89
LHR-LDA^*ξ*^	**99.47**	87.38	95.00	**99.44**	**98.75**	**99.47**	97.09	95.42	**99.47**	96.83
LHR-NB^*ξ*^	97.79	90.32	92.21	97.24	98.61	97.24	**98.04**	89.77	97.79	95.44
LHR-KNN^*ξ*^	98.45	91.94	96.10	91.00	*100*	*100*	*99.02*	94.32	99.45	96.70
LHR-HKNN^*ξ*^	*100*	90.32	**97.40**	97.40	*100*	100	97.06	94.32	100	**97.39**
I-RELIEF-SVM^*η*^[12]	83.43	75.81	92.21	92.21	94.44	83.98	88.24	82.95	81.12	86.04
I-RELIEF-LDA^*η*^[12]	81.17	74.05	89.46	89.46	92.86	80.06	80.64	87.50	80.18	83.93
I-RELIEF-NB^*η*^[12]	85.08	67.74	84.42	84.42	91.67	86.74	73.53	81.82	87.29	82.52
I-RELIEF-KNN^*η*^[12]	88.4	82.26	96.10	96.10	94.44	88.40	91.18	86.36	87.85	90.12
I-RELIEF-HKNN^*η*^[12]	83.98	77.42	96.10	96.10	95.83	86.16	85.29	77.27	83.98	86.90

^***ξ***^Classification with our selected genes.

^***η***^Classification with selected genes by [11].

^***γ***^The value of 96.97 in [26] could have been rounded to 97.00 and is suboptimal.

The optimal and suboptimal values on each tested data are highlighted in bold and italic, respectively. The averaged performance of the proposed method with HKNN classifier is suboptimal to BMSM-SVM by a neglectable difference. Besides, the averaged performance of LHR, coupling with five classifiers show a dramatically smaller variance (0.725) than other BMSM does (2.191), thus implying a high capability of stability with respect to classification models.

For the individual dataset, LHR outperformed or achieved comparable performance to the best result reported in the literature. For the CNS data, the LHR-SVM, LHR-LDA and LHR-HKNN achieved superior performances with almost 100*%* accuracy, which is much higher than the second best performance by k-TSP [19]. For the colon data, although the accuracy of the LHR-based classifier is worse than that of BMSF-SVM, IVGA-SVM and LOGO, the accuracy of all the five classifiers are similar. This implies that the selected genes are very robust to the choice of different classifiers. Similar results are observed on the DLBCL, prostate2 and prostate3 datasets. For the GCM, leukemia, lung and prostate1 datasets, the LHR-based classifier was ranked either first or second. The selected genes tested by the five classifiers show similar performance on the leukemia, lung and prostate1 datasets. For the prostate2 data, BMSF-SVM realized remarkably good accuracy, although the results using the other three classifiers with BMSF feature selection are less impressive. LOGO also performed nicely, yet the average is suboptimal to LHG. In comparison, the performance using LHR feature selection is fairly stable. For the prostate3 data, LOGO based classifiers performed very well, while the LHR based ones were slightly less accurate than the top ones. Compared with LOGO in terms of the ability to select informational genes, the proposed algorithm achieved comparable performance by reaching the classification accuracy of 97.39*%*, which is slightly less than LOGO of 97.61*%*.

When considering the average accuracy for each algorithm across all cancers datasets, the top four methods with the highest average accuracy are LOGO-HKNN, BMSF-SVM, LHR-KNN/LOGO-KNN, LHR-SVM and LHR-HKNN. The proposed scheme has a slightly lower average accuracy than BMSF-SVM and LOGO-HKNN, but a higher accuracy than the others. However, the values for *mean* ± *standarddeviation* of the averaged accuracy are 96.65±0.725 for LHR, 97.61±1.5 for LOGO and 94.88±2.191 for BMSF. This shows that the proposed LHR outperforms both LOGO and BMSF in terms of overall accuracy as well as confirming its excellent stability in terms of the choice of classification method.

### Comparison with standard feature selection methods

For comparison with other feature selection models, eleven standard techniques were tested as well as the proposed LHR. The selected techniques include *t*-statistic (*t*-stat), twoing rule (TR), information gain (IG), Gini index (Gini), max minority (MaxM), sum minority (SumM), sum of variances (SumV), one-dimensional support vector machine (OSVM), minimum redundancy maximum relevance (mMRM) [27] and I-RELIEF [28]. The code for the first eight schemes is available through RankGene at http://genomics10.bu.edu/yangsu/rankgene. The code for mRMR is available at http://penglab.janelia.org/proj/mRMR/, where two implementations of mRMR: namely, MID and MIQ, are provided. The I-RELIEF package is available at http://plaza.ufl.edu/sunyijun/[28].

It has been suggested by the author in [25,27] that accurate discretization could improve the performance of mRMR. The author also reported consistent results when the expression values are transformed into 2 or 3 states using *μ*±*kσ* with *k* ranging from 0.5 to 2, and where *μ* and *σ* are gene specific mean and standard deviation, respectively (http://penglab.janelia.org/proj/mRMR/FAQ_mrmr.htm). In our experiments, we followed the transformation rule suggested in [25] to simplify the comparison. Expression values greater than *μ*+*σ* were set to 1; values between *μ*-*σ* and *μ*+*σ* were set to 0; and values less than *μ*-*σ* were set to -1.

In each experiment, a feature selection scheme was first used to select the informative genes, followed by classification tests on the truncated dataset. For subjective comparison, we set the number of informative genes for the selected feature selection scheme to be the same as that determined by LHR, which usually finds a relatively small number of genes (less than 30). This allowed us to examine whether the limited number of informative genes generated by LHR had more discriminative power than those generated by the other methods.

**Figure 2:**
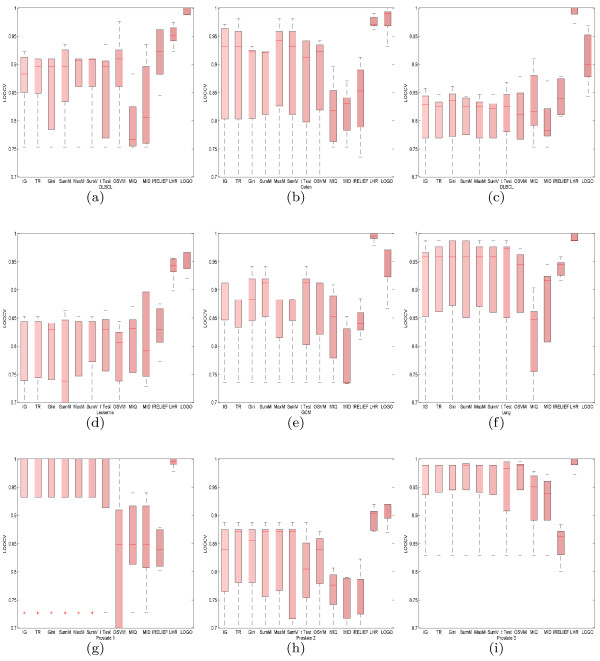
**Performance comparison of the LHR with 12 standard feature selection schemes (FSSs).** Nine benchmarked microarray datasets **(a-i)**, whose name was positioned in middle below of the x-axis, were used to test the performance of the FSSs. For each tested data, the results after five classification, coupling with 13 FSSs through LOOCV are box plotted. The proposed LHR outperformed or archived comparable performance to other methods. Moreover, the results after LHR show small variation on LOOCV error when tested with different classifiers, implying a high degree of robustness.

The LOOCV accuracy for each of the five classification algorithms (LDA, NB, SVM, KNN, and HKNN) is reported in Table 5. The number of genes selected by LHR is listed in the second column and the same number is used to create the truncated data for the other feature selection schemes. In most cases, the variables selected by LHR achieved the optimal or suboptimal LOOCV accuracy when coupled with the five classifiers. To investigate the extent of the information conveyed by the selected genes, we created a box plot of the LOOCV accuracy for the five classification algorithms (LDA, SVM, KNN, NB, and HKNN) on each of the tested datasets in Figure 2. A remarkable characteristics of the proposed LHR is its low dependence on the classifiers, resulting in the corresponding box plot having a narrower bandwidth than that for the other methods, shown in Figure 2. This property implies that the genes selected by LHR are highly informative, and thus the discriminative performance is robust to the choice of different classifiers.

**Table 5:** Performance comparison of the LHR with 12 standard feature selection schemes (FSSs)

**Classifier**	**FSS**	**DLBCL**	**Prostate1**	**GCM**	**Prostate2**	**CNS**	**Leukemia**	**Prostate3**	**Colon**	**Lung**	**Avg.**
	**No. genes**	**27**	**22**	**6**	**24**	**7**	**23**	**6**	**18**	**5**	
	IG	93.5	96.1	81.8	84.1	88.2	97.2	*100*	87.1	99.4	91.9
	TR	94.8	**97.1**	82.1	84.1	85.3	97.2	*100*	**91.9**	99.4	92.4
	Gini	93.5	95.1	80.7	81.8	88.2	95.8	*100*	83.9	99.4	90.9
	SumM	94.8	93.1	81.8	70.5	85.3	98.6	*100*	88.7	98.9	90.2
	MaxM	94.8	**97.1**	82.1	84.1	85.3	97.2	*100*	**91.9**	99.4	92.4
SVM^*η*^	SumV	94.8	**97.1**	82.1	84.1	85.3	97.2	*100*	**91.9**	99.4	92.4
	*t*-stat	92.2	91.2	83.2	81.8	82.4	95.8	*100*	87.1	98.9	90.3
	OSVM	**98.7**	93.1	80.7	73.9	85.3	95.8	72.7	85.5	98.3	87.1
	MID^*ξ*^	75.3	75.3	75.3	75.3	73.5	65.3	72.7	64.5	82.9	73.4
	MIQ^*ξ*^	75.3	75.3	75.3	75.3	73.5	65.3	72.7	64.5	82.9	73.4
	I-RELIEF	92.2	88.2	81.2	83.0	83.4	94.4	81.2	75.8	84.0	84.8
	LHR	94.8	96.1	*100*	*95.5*	*100*	98.6	*100*	**87.1**	*100*	**96.9**
	LOGO	*100*	*100*	**88.9**	**92.0**	**97.1**	*100*	*100*	*91.9*	*100*	*96.7*
	IG	88.2	90.1	82.9	79.6	88.3	92.0	*100*	80.5	97.3	88.8
	TR	88.0	90.1	82.9	80.3	86.7	93.0	*100*	82.6	97.8	89.0
	Gini	79.5	90.2	83.6	79.9	88.3	94.5	*100*	82.6	98.3	88.5
	SumM	86.1	92.1	82.5	71.9	89.2	91.6	*100*	79.3	98.3	87.9
	MaxM	90.7	94.2	82.9	80.7	84.2	94.3	*100*	80.7	97.8	89.5
LDA	SumV	90.9	91.2	82.1	84.0	88.3	92.9	*100*	74.0	97.3	89.0
	*t*-stat	77.5	89.4	83.9	84.2	82.5	91.6	97.5	80.5	93.4	86.7
	OSVM	**97.4**	92.3	79.6	84.4	85.0	92.9	40.0	82.4	98.9	83.7
	MID^*ξ*^	75.5	83.9	81.6	83.9	**90.8**	78.9	84.2	77.6	96.7	83.7
	MIQ^*ξ*^	76.3	79.3	78.2	72.9	73.3	85.9	83.3	78.8	95.6	80.4
	I-RELIEF	89.5	80.6	80.7	87.5	81.2	92.9	80.2	74.0	80.1	83.0
	LHR	95.0	**97.1**	*99.4*	**95.4**	**99.5**	**98.8**	**99.5**	*87.4*	**99.5**	*96.8*
	LOGO	*98.6*	*98.0*	**90.0**	*95.6*	86.7	*100*	*100*	**86.9**	*100*	**95.1**
	IG	88.3	93.1	80.0	84.1	91.2	95.8	*100*	87.1	98.9	90.9
	TR	89.6	93.1	80.0	84.1	88.2	95.8	*100*	88.7	98.9	90.9
	Gini	89.6	92.2	80.4	83.0	91.2	95.8	*100*	88.7	98.9	91.1
	SumM	89.6	92.2	80.7	73.9	91.2	95.8	*100*	88.7	98.9	90.1
	MaxM	89.6	93.1	80.0	84.1	88.2	95.8	*100*	88.7	98.9	90.9
NB	SumV	89.6	93.1	80.0	84.1	88.2	95.8	*100*	88.7	98.9	90.9
	*t*-stat	89.6	94.1	82.5	83.0	91.2	98.6	*100*	79.0	98.3	90.7
	OSVM	90.9	94.1	81.1	81.8	91.2	95.8	*100*	83.9	98.3	90.8
	MID^*ξ*^	76.6	76.6	80.5	75.3	88.2	84.7	84.8	80.6	97.8	82.8
	MIQ^*ξ*^	80.5	83.1	77.9	79.2	73.5	94.4	84.8	74.2	97.2	82.8
	I-RELIEF	84.4	73.5	87.3	81.8	85.1	91.7	87.3	67.7	86.7	82.8
	LHR	**92.2**	*98.0*	*97.2*	89.8	**97.8**	98.6	97.8	*90.3*	97.2	*95.4*
	LOGO	*98.7*	93.1	84.3	*94.3*	97.1	*100*	*100*	*90.3*	*100*	**95.3**
	IG	92.2	96.1	85.7	84.1	91.2	98.6	*100*	88.7	98.9	92.8
	TR	90.9	98.0	84.6	84.1	88.2	98.6	*100*	87.1	98.9	92.3
	Gini	90.9	92.2	86.1	84.1	88.2	98.6	*100*	85.5	98.9	91.6
	SumM	93.5	92.2	84.3	86.4	94.1	98.6	*100*	87.1	98.9	92.8
	MaxM	90.9	**98.0**	84.6	84.1	88.2	98.6	*100*	87.1	98.9	92.3
KNN^*η*^	SumV	90.9	**98.0**	84.6	84.1	88.2	98.6	*100*	87.1	98.9	92.3
	*t*-stat	93.5	94.1	86.8	86.4	91.2	97.2	*100*	88.7	99.4	93.0
	OSVM	90.9	93.1	87.9	80.7	91.2	94.4	84.8	85.5	98.9	89.7
	MID ^*ξ*^	88.3	89.6	90.9	87.0	85.3	90.3	93.9	77.4	91.2	88.2
	MIQ ^*ξ*^	93.5	87.0	87.0	89.6	85.3	91.7	93.9	79.0	91.2	88.7
	I-RELIEF	96.1	91.2	87.8	86.4	88.4	94.4	87.8	82.3	88.4	89.2
	LHR	96.1	*99.0*	*100*	94.3	**99.4**	*100*	99.4	**91.9**	*100*	*97.8*
	LOGO	*100*	*99.0*	**94.6**	96.6	**94.1**	*100*	*100*	91.9	*100*	**97.4**
	IG	90.9	97.1	83.9	85.2	91.2	95.8	100	83.9	98.9	91.9
	TR	90.9	95.1	82.5	85.2	88.2	97.2	*100*	87.1	98.9	91.7
	Gini	90.9	93.1	84.3	84.1	94.1	98.6	*100*	87.1	98.9	92.3
	SumM	92.2	91.2	83.9	84.1	91.2	98.6	*100*	87.1	*100*	92.0
	MaxM	90.9	95.1	82.5	85.2	88.2	97.2	*100*	87.1	98.9	91.7
HKNN ^*η*^	SumV	90.9	95.1	82.5	85.2	88.2	97.2	*100*	87.1	98.9	91.7
	*t*-stat	89.6	91.2	81.4	81.8	94.1	97.2	*100*	83.9	99.4	91.0
	OSVM	89.6	92.2	83.9	79.5	91.2	97.2	87.9	87.1	99.4	89.8
	MID ^*ξ*^	80.5	81.8	87.0	83.1	79.4	84.7	90.9	79.0	95.0	84.6
	MIQ ^*ξ*^	88.3	83.1	80.5	89.6	82.4	91.7	90.9	75.8	93.9	86.2
	I-RELIEF	96.1	85.3	84.0	77.3	84.0	95.8	84.0	77.4	86.2	85.6
	LHR	**97.4**	**97.1**	**100**	**94.3**	*100*	*100*	*100*	**90.3**	**100**	**97.7**
	LOGO	*100*	*99.0*	**96.8**	*96.6*	97.1	*100*	*100*	91.9	*100*	*97.9*

^***ξ***^Preprocessing of the data via *t*-test with confidence leel of 0.01 to reduce the computation burden on estimating of mutual information.

^***η***^Hyper-parameters are estimated via 5-fold cross validation.

The number of genes is determined by LHR and used for all other FSSs. LOOCV criteria is used to evaluate the performance of the FSSs, coupling with five classification models. The optimal and suboptimal accuracy (columnwise) on each tested data are highlighted in bold and italic, respectively.

### Computation complexity

Solving of the LHR algorithm involves in a quadratic minimization problem (Eq. (4)) for each sample. Therefore, it needs a much higher computational cost than linear method does, such as I-RELIEF and LOGO. Although the matrix of ***H***^*T*^***WH*** in Eq. (4) is positive-definite and in small size, the minimization problem of Eq. (4) can be solved in polynomial time (*O*(*n*^3^) for *n* NNs of a sample). Thus, the complexity in each iteration are approximately *O*(*n*^3^∗*N*) times higher than I-RELIEF does.

## Conclusions

In this paper, we proposed a new feature weighting scheme to overcome the common drawbacks of the RELIEF family. The nearest miss and hit subsets are approximated by constructing a local hyperplane. Then feature weight updating is achieved by measuring the margin between the sample and its hyperplane in a general RELIEF framework. The main contribution of the new variation is that the margin is more robust to the noise and outliers than those of earlier works. Therefore, the feature weights can characterize the local structure more accurately. Experimental results on both synthetic and real-world microarray datasets validated our findings when combining the proposed method with five classic classifiers. The performance of the proposed weighting scheme performed is superior in terms of classification error on most test datasets. Extensive experiments demonstrated that the proposed scheme has three remarkable characteristics: 1) high accuracy in classification, 2) excellent robustness to noise and 3) good stability with respect to various classification algorithms.

## Methods

### RELIEF

The RELIEF algorithm has been successfully applied in feature weighting owing to its simplicity and effectiveness [12,13]. The main idea of RELIEF is the iterative adjustment of feature weights according to their ability to discriminate among neighboring patterns. Mathematically, suppose that ***X***={***x***_1_,***x***_2_,⋯,***x***_*n*_}_*d*×*N*_ is a randomly selected sample matrix of binary class data where each sample ***x*** has *d* dimensions, ***x***={*x*_1_,*x*_2_,⋯,*x*_*d*_}. One can estimate the two nearest neighbors, where one is from the same class (called *the nearest hit* or NH) and the other is from a different class (called *the nearest miss* or NM). Then, weight *w*_*f*_ of the *f*-th (*f*=1,2,⋯,*d*) feature is updated by the heuristic estimation:

(1) $$w_{f}\leftarrow w_{f}+\left|x_{f}-{\text{NM}}_{f}\right|-\left|x_{f}-{\text{NH}}_{f}\right|$$

where *NM*_*f*_,*NH*_*f*_ denote the *f*-th coordinate value of vector *NM* and *NH*, respectively. Since no exhaustive or iterative search is needed for RELIEF updates, this scheme is very efficient in processing data with huge dimensions. Thus, it is particularly promising for large-scale problems such as analysis of microarray data [3,12,27]. The author generalized the updates scheme to compute the maximum expected margin **E**[ *ρ*(***w***)] by scaling the features [11,12] to overcome the drawbacks of RELIEF, such as outlier detection and inaccurate updates:

(2) $$\;\begin{array}{c} \text{E}[\;\rho (w)]≐w^{T}\;\left(\underset{x_{n}\in \text{NM}(x_{i})}{\text{E}}\;\left[\left|x_{n}-x_{i}\right|\right]-\;\;\underset{x_{n}\in \text{NH}(x_{i})}{\text{E}}\;\left(\left|\left[x_{n}-x_{i}\right)\right|\right)\;\right)\\ \;=w^{T}\;\left(\underset{x_{n}\in \text{NM}(x_{i})}{\sum }P\left(x_{n}=\text{NM}\left(x_{i}\right)|w\right)\left|x_{n}-x_{i}\right|\right)\\ \;\left(-\underset{x_{n}\in \text{NH}(x_{i})}{\sum }P\left(x_{n}=\text{NH}\left(x_{i}\right)|w\right)\left|x_{n}-x_{i}\right|\right)\\ \;=w^{T}z_{n} \end{array}$$

with $z_{n}=\underset{x_{n}\in \text{NM}(x_{i})}{\sum }P(x_{n}=\text{NM}(x_{i})|w)|x_{n}-x_{i}|-\underset{x_{n}\in \text{NH}(x_{i})}{\sum }P(x_{n}=\text{NH}(x_{i})|w)|x_{n}-x_{i}|$, where *NM*(***x***_*i*_)={***x***_*n*_:1≤*n*≤*N*,*y*_*i*_≠*y*_*n*_} and *NH*(***x***_*i*_)={***x***_*n*_:1≤*n*≤*N*,*y*_*i*_=*y*_*n*_} are index sets of the nearest miss and the nearest hit for the sample ***x***_*i*_. *N* is the sample size. *P*(***x***_*n*_=*NM*(***x***_*i*_)|***w***) (or *P*(***x***_*n*_=*NH*(***x***_*i*_)|***w***)) is the probability of a sample ***x***_*n*_ being in the set of *NM*(***x***_*i*_) (or *NH*(***x***_*i*_)) in the feature space scaled by weights ***w***. Though the probability distributions are initially unknown, they can be estimated through kernel density estimation [29]. The authors called this method I-RELIEF and showed that it achieved significant performance improvement over the traditional models. Classification of a feature scaled dataset achieved higher accuracy than standard techniques such as the SVM [1,2,30] and NN model [31]. Feature weighting is also robust to noisy features. To obtain a sparse and economic feature weighting, Sun incorporated the *l*_1_ penalty into the optimization of I-RELIEF and named the algorithm by Logo (*fit locally and think globally*) [12]. Extensive experiments have demonstrated that Logo could accurately grasp the intrinsic structure of the data and match nicely with classic classification models.

However, the expectation in Eq. (2) is obtained by averaging the nearest neighbors. Therefore, feature weight estimation may be less accurate if the samples contain many outliers or most of the features are irrelevant. In both cases, the distance between the tested sample and its nearest neighbor is a large value. It follows that large bias is introduced to margin estimation by using the such averaging operation. Although the influence of abnormal samples can be reduced by introducing kernel distribution estimation [11,12], this in turn introduces additional free parameters. Moreover, probability estimation via kernel approximation is sensitive to the sample size [28]. Therefore, it limits the empirical applications such as analysis of microarray data, which the data are notoriously known for the fact that the dimension of the sample observations is much smaller than that of the sample features [32]. In this paper, we propose using a local hyperplane to approximate the set of the nearest hit and miss, and then estimate the feature weight by maximizing the expected margin defined by the hyperplane. The advantage of this approximation is that the hyperplane is more robust to noisy feature degradation than averaging all the neighbors [11-13].

### Local hyperplane conditional on feature weight

Processing high-dimensional data by mapping the data of interest into an embedded non-linear manifold within the higher-dimensional space has attracted wide interest in machine learning. The local hyperplane approximation shares similar merits with local linear embedding methods [12,26,33]. It assumes that the samples’ structure is locally linear and therefore each sample lies on a local linear hyperplane, spanned by its nearest neighbors. Mathematically, let us assume that the feature weights $w≐\{w_{1},w_{2},\cdots \;,w_{I}\}$ are known in advance. Thus, sample ***x*** can be represented by a local hyperplane of class *c*, conditional on the feature weight ***w***, as:

(3) $${\text{LH}}_{c}(x)=\{s\;|\;s=WH\alpha \},$$

where ***H*** is an *I*×*n* matrix comprising *n* NNs of sample ***x***: ***H***={***h***_1_,***h***_2_,⋯,***h***_*n*_}, with ***h***_*i*_ being the *i*-th nearest neighbor (called the *prototype*) of class *c*. ***W*** is a diagonal matrix with diagonal element *w*_*i*_ being the weight of the *i*-th feature. The parameters of ***α***=(*α*_1_,…,*α*_*n*_)^*T*^ are the weights of the prototypes {***h***_*i*_, *i*=1,2,…,*n*}. These can be viewed as the spanning coefficients of subspace *LH*_*c*_(***x***). Therefore, the hyperplane can be represented as: {· |***Hα***=*α*_1_***Wh***_1_+*α*_2_***Wh***_2_+…+*α*_*n*_***Wh***_*n*_}. The projection *LH*_*c*_(***x***) of ***x*** onto the hyperplane can be computed by minimizing the distance between sample *x* and the hyperplane, both of which are dependent on the feature weight. Therefore, the value of ***α*** can be estimated as:

(4) $$\;\begin{array}{c} J_{c}(\alpha )=\;\arg\underset{\alpha }{\min}\left\{\frac{1}{2}\overset{I}{\underset{i=1}{\sum }}{\left(w_{i}x_{i}-s_{i}\right)}^{2}+\lambda \overset{n}{\underset{j=1}{\sum }}\alpha_{i}^{2}\right\}\\ \;=\arg\underset{\alpha }{\min}\left\{\frac{1}{2}{\left(x-H\alpha \right)}^{T}W\left(x-H\alpha \right)+\lambda \alpha^{T}\alpha \right\}\\ \text{Subject to}\\ \;\overset{k}{\underset{i=1}{\sum }}\alpha_{i}=1,\;\alpha \geq 0 \end{array}$$

The regularization parameter *λ* is used to emphasize the “smoothing” effect of the optimum solution, which degenerates to be an unit vector in certain radical cases.

We propose using a hyperplane to represent the set of the nearest miss *NM*(***x***) and nearest hit *NH*(***x***) for a given sample ***x***. The advantage of the representation is the robust characterization of the local sample patterns. Then the distances between the sample and its *NH* (or *NM*) set can be estimated from the local hyperplane rather than averaging across all samples within the set. Therefore, we redefine the margin for a sample ***x*** as $\rho_{n}≐d(x_{n}-{\text{LH}}_{\text{NM}}(x_{n}))-d(x_{n}-{\text{LH}}_{\text{NH}}(x_{n}))$. The feature weights are then estimated by maximizing the total margin:

(5) $$\;\begin{array}{c} \underset{w}{\max}\text{E}[\;\rho (w)]=\frac{1}{N}\underset{w}{\max}\overset{N}{\underset{n=1}{\sum }}\left(\overset{I}{\underset{i=1}{\sum }}\omega_{i}\left|x_{n}^{\left(i\right)}-{\text{LH}}_{\text{NM}}^{\left(i\right)}\left(x_{n}\right)\right|\right)\\ \;\left(-\overset{I}{\underset{i=1}{\sum }}\omega_{i}\left|x_{n}^{\left(i\right)}-{\text{LH}}_{\text{NH}}^{\left(i\right)}\left(x_{n}\right)\right|\right)\\ \;=\underset{w}{\max}w^{T}\frac{1}{N}\overset{N}{\underset{n=1}{\sum }}\left(\overset{I}{\underset{i=1}{\sum }}\left|x_{n}^{\left(i\right)}-\alpha H_{\text{NM}}^{\left(i\right)}\left(x_{n}\right)\right|\right)\\ \;\left(-\overset{I}{\underset{i=1}{\sum }}\left|x_{n}^{\left(i\right)}-\beta H_{\text{NH}}^{\left(i\right)}\left(x_{n}\right)\right|\right)\\ \;=\underset{w}{\max}w^{T}z_{n} \end{array}$$

where vector ***z***_*n*_ is defined as: $z_{n}=\frac{1}{N}\overset{N}{\underset{n=1}{\sum }}\left(\overset{I}{\underset{i=1}{\sum }}|x_{n}^{\left(i\right)}-\alpha H_{\text{NM}}^{\left(i\right)}(x_{n})|-\overset{I}{\underset{i=1}{\sum }}|x_{n}^{\left(i\right)}-\beta H_{\text{NH}}^{\left(i\right)}(x_{n})|\right)$, where ***H***_*NM*_(***x***_*n*_) and ***H***_*NH*_(***x***_*n*_) are the nearest neighbors of the set of the nearest miss and hit of sample ***x***_*n*_. ***α***_*n*_ and ***β***_*n*_ are the coefficients for spanning hyperplane ${\text{LH}}_{\text{NM}}^{\left(n\right)}$ and ${\text{LH}}_{\text{NH}}^{\left(n\right)}$. ***w*** is a vector with its *i*-th element ***w***(*i*) being the weight of the *i*-th feature, for *i*=1,2,…,*I*. To solve the minimization problem of Eq. (5), the parameters of ***α***_*n*_, ***β***_*n*_, which are dependent on the nearest neighbors, must be estimated. The main problem with this estimation, however, is that the nearest neighbors of a given sample are unknown before learning. In the presence of many thousands of irrelevant features, the nearest neighbors defined in the original space can be completely different from those in the induced space. Therefore, the nearest neighbors defined in the original feature space may not be the same in the weighted feature space. To address these difficulties, we use an iterative algorithm, similar to the Expectation Maximization algorithm and I-RELIEF [11], to estimate the feature weights. The detailed numerical solution is provided in Additional file 1: S.1. The pseudo-code for LH-RELIEF is summarized in Additional file 2: S.2.

## Availability of supporting data

The Matlab code used to tested on the Fermat’s spiral and the cancer microarray datasets is available at http://sunflower.kuicr.kyoto-u.ac.jp/\~{r}uan/LHR/.

## Competing interests

The authors declare that they have no competing interests.

## Authors’ contributions

HM designed the LHR algorithm, participated in the numerical experiments and drafted the manuscript. PY participated in the numerical experiments. MN participated in the design of the study and TA participated in the study design and helped to draft the manuscript. All authors read and approved the final manuscript.

## Supplementary Material

Additional file 1 **S.1.** Numerical solution for LHR. Click here for file

Additional file 2 **S.2.** Pseudo-code for LHR. Click here for file
