# Supplementary material for: Feature weight estimation for gene selection: a local hyperlinear learning approach
Source: BMC Bioinformatics. 2014 Mar 14;15:70. doi: 10.1186/1471-2105-15-70 (PMC4007530; doi:10.1186/1471-2105-15-70)
Supplement: Additional file 1 — S.1. Numerical solution for LHR. [file 1471-2105-15-70-S1.pdf]

S.1: Numerical Solution

Step 1: In i-th iteration, for a given sample $\boldsymbol{x}$, we estimate the parameter of $\boldsymbol{\alpha}$ by constructing the local hyperplane of the nearest hit set within induced feature space. It is trivial to show that the minimization of Eq. (5) is equivalent to solving the following quadratic programming:

$$\begin{aligned} &\min_{\boldsymbol{\alpha}} \frac{1}{2}\boldsymbol{\alpha}^T \bar{\boldsymbol{H}} \boldsymbol{\alpha} + \boldsymbol{f}^T \boldsymbol{\alpha} \\ &s.t. \quad \mathbf{1}^T \boldsymbol{\alpha} = 1, \ \boldsymbol{\alpha} \geq 0 \end{aligned} \tag{S.1.6}$$

where $\bar{\boldsymbol{H}} = \boldsymbol{H}^T \boldsymbol{W}^{(i)} \boldsymbol{H}$, $\boldsymbol{f} = -\boldsymbol{x}^T \boldsymbol{W}^{(i)} \boldsymbol{H}$, and $\mathbf{1}$ is an unitary vector whose elements are all being 1. The matrix of $W^{(i)}$ is the i-th feature weight matrix, satisfying $W^{(i)}\mathbf{1} = \boldsymbol{w}$. The parameter of $\boldsymbol{\beta}$ for nearest miss hyperplane is obtained similarly. Minimization of Eq. (S.1.6) is a constrained quadratic program problem and standard techniques can be used to obtain its solution. In particular, since the matrix of $\bar{\boldsymbol{H}}$ is symmetric and non-negative, the minimization could be solved efficiently through standard techniques, such as active set.

Step 2: Estimation of the total margin with respect to $\boldsymbol{w}^{(i)}$.

$$\begin{aligned} \rho(\boldsymbol{w}^{(i)}) &= \frac{1}{N}\sum_{n=1}^{N}(\sum_{i=1}^{I}\omega_i|\boldsymbol{x}_n^{(i)} - \boldsymbol{\alpha}\boldsymbol{H}_{NM}^{(i)}(\boldsymbol{x}_n)| \\ &- \sum_{i=1}^{I}\omega_i|\boldsymbol{x}_n^{(i)} - \boldsymbol{\beta}\boldsymbol{H}_{NH}^{(i)}(\boldsymbol{x}_n)|) \end{aligned} \tag{S.1.7}$$

Step 3: Estimation of the weight $\boldsymbol{W}$ in $(i+1)$-th iteration.

$$\begin{aligned} \boldsymbol{w} &= \arg\max_{\boldsymbol{w}} \rho(\boldsymbol{w}^{(i)}) \\ &= \arg\max_{\boldsymbol{w}} \{\frac{1}{N}\sum_{n=1}^{N}(\sum_{i=1}^{I}\omega_i|\boldsymbol{x}_n^{(i)} \\ &- \boldsymbol{\alpha}\boldsymbol{H}_{NM}^{(i)}(\boldsymbol{x}_n)| - \sum_{i=1}^{I}\omega_i|\boldsymbol{x}_n^{(i)} - \boldsymbol{\beta}\boldsymbol{H}_{NH}^{(i)}(\boldsymbol{x}_n)|)\} \end{aligned} \tag{S.1.8}$$

The above steps iterate alternatively until their convergence. The last two steps are similar to the one used in I-RELIEF, and we name our scheme as **LHR** since it requires a local hyperplane approximation.
