# Supplementary material for: Feature weight estimation for gene selection: a local hyperlinear learning approach
Source: BMC Bioinformatics. 2014 Mar 14;15:70. doi: 10.1186/1471-2105-15-70 (PMC4007530; doi:10.1186/1471-2105-15-70)
Supplement: Additional file 2 — S.2. Pseudo-code for LHR. [file 1471-2105-15-70-S2.pdf]

S.2: Pseudo-code for LHR

Algorithm 1: LHR ALGORITHM(V, W, λ)

comment: Variables initialization: $\boldsymbol{w} = \frac{1}{I}$, stopping criteria ϵ, number of iterations T

for $t \leftarrow 1$ **to** T

while $\|\boldsymbol{w}^{(t+1)} - \boldsymbol{w}^{(t)}\| > \epsilon$

do
1. Estimate the coefficients for hyperplane of nearestmiss and hit $\boldsymbol{\alpha}, \boldsymbol{\beta}$;
2. Calculate the margin by Eq .S.1.7, conditional on the estimated feature weights in t step;
3. Update the weights by Eq. S.1.8, conditional on the estimated hyperplane coefficients.

return $(\boldsymbol{w})$
